# Value of information analysis in telehealth for chronic heart failure management

**DOI:** 10.1371/journal.pone.0218083

**Published:** 2019-06-20

**Authors:** Andrija S. Grustam, Nasuh Buyukkaramikli, Ron Koymans, Hubertus J. M. Vrijhoef, Johan L. Severens

**Affiliations:** 1 Erasmus School of Health Policy & Management, Erasmus University Rotterdam, Rotterdam, the Netherlands; 2 Professional Health Solutions & Services Department, Philips Research, Eindhoven, the Netherlands; 3 Institute of Medical Technology Assessment, Erasmus University Rotterdam, Rotterdam, the Netherlands; 4 Department of Patient & Care, Maastricht UMC, Maastricht, the Netherlands; 5 Department of Family Medicine and Chronic Care, Vrije Universiteit Brussels, Brussels, Belgium; 6 Panaxea b.v., Amsterdam, the Netherlands; US Army Engineer Research and Development Center, UNITED STATES

## Abstract

**Objectives:**

Value of information (VOI) analysis provides information on opportunity cost of a decision in healthcare by estimating the cost of reducing parametric uncertainty and quantifying the value of generating additional evidence. This study is an application of the VOI methodology to the problem of choosing between home telemonitoring and nurse telephone support over usual care in chronic heart failure management in the Netherlands.

**Methods:**

The expected value of perfect information (EVPI) and the expected value of partially perfect information (EVPPI) analyses were based on an informal threshold of €20K per quality-adjusted life-year. These VOI-analyses were applied to a probabilistic Markov model comparing the 20-year costs and effects in three interventions. The EVPPI explored the value of decision uncertainty caused by the following group of parameters: treatment-specific transition probabilities between New York Heart Association (NYHA) defined disease states, utilities associated with the disease states, number of hospitalizations and ER visits, health state specific costs, and the distribution of patients per NYHA group. We performed the analysis for two population sizes in the Netherlands—patients in all NYHA classes of severity, and patients in NYHA IV class only.

**Results:**

The population EVPI for an effective population of 2,841,567 CHF patients in All NYHA classes of severity over the next 20 years is more than €4.5B, implying that further research is highly cost-effective. In the NYHA IV only analysis, for the effective population of 208,003 patients over next 20 years, the population EVPI at the same informal threshold is approx. €590M. The EVPPI analysis showed that the only relevant group of parameters that contribute to the overall decision uncertainty are transition probabilities, in both All NYHA and NYHA IV analyses.

**Conclusions:**

Results of our VOI exercise show that the cost of uncertainty regarding the decision on reimbursement of telehealth interventions for chronic heart failure patients is high in the Netherlands, and that future research is needed, mainly on the transition probabilities.

## Introduction

Economic evaluation, or cost-effectiveness analysis, resorts to modeling in order to analyze costs and outcomes of technology implementation in healthcare, synthesize different types of data, and extrapolate short term trial results to longer term. Historically those analytical models were deterministic only, but due to irrelevance of p-values and inference in medical decision making [1], the probabilistic models were developed and the Probabilistic Sensitivity Analysis (PSA) emerged to represent parameter uncertainty. PSA is executed by assigning each uncertain input parameter in the analysis a plausible distribution, and sampling each input parameter from their assigned distributions simultaneously [2, 3]. The incremental PSA results can be presented in cost-effectiveness planes, where the incremental result of each simulation iteration in the PSA is plotted, and the ‘cloud’ of results would be interpreted together with relevant Willingness-to-Pay (WTP) thresholds to give an estimate of the probability of being cost-effective and the associated uncertainty around the incremental cost and effect results. Those PSA results for different thresholds were then represented by Cost-effectiveness Acceptability Curves (CEACs) [4] and cost-effectiveness frontiers [5]. However, the CEACs, although being useful in understanding the uncertainty of the cost-effectiveness of alternative interventions, did not provide any insight into the decision uncertainty and do not locate where the uncertainty of the decision originated from. Thus, the Value of Information (VOI) analysis gained traction in economic evaluation in healthcare [6–8].

### Value of information analysis in healthcare

VOI analysis provides information on opportunity cost of a decision in healthcare [9]. In the cost-effectiveness analysis the preferred scenario is the one with the maximum expected net benefit of the intervention, either Net Monetary Benefit (NMB), which is the costs borne by the therapy, or Net Health Benefit (NHB), usually expressed in Quality Adjusted Life Years (QALYs). Expected net benefit is defined as the mean of the net benefits across all model iterations [10]. VOI is a Bayesian analytical framework which concerns itself with identification and adoption of the alternative with the maximum expected net benefit and recognizes that such decisions are surrounded by uncertainty which cannot be expressed via p-values [10]. The uncertainty about the alternatives results in wrong decision being made, with opportunity costs. The expected cost of the wrong decision is based on the probability that the wrong decision will be made, and the size of the loss with the wrong decision. Expected Value of Perfect Information (EVPI) analysis is useful because CEACs provide only the probability of being cost-effective and EVPI determines sort of expected cost of uncertainty, which is determined jointly by the probability that a decision based on existing information will be wrong and the consequences of a wrong decision. The Expected Value of Partially Perfect Information (EVPPI) analysis pinpoints a parameter or parameter group, which contributes to the parametric uncertainty most. Thus, the VOI analysis informs decision makers how large the cost of a wrong decision is and whether it is cost-effective to conduct further research on model parameters to lower the uncertainty in the decision-making process [7].

VOI analysis provides insights into the maximum that authorities should pay for further research (i.e., EVPI). EVPI is possibly the best measure of uncertainty surrounding a particular decision in CEA [11]. However, both EVPI and EVPPI do not include methodological and structural uncertainty, only the parameter uncertainty. Methodological uncertainty arises when “there are different normative views about what constitutes the correct approach for optimum decision making” [12], e.g., discount rates or time horizon in the analysis [3]. It also includes the perspective taken (e.g., provider, payer, societal), how health gains are valued, e.g., via preference-based or non-preference-based methods [13], types of disease outcomes (e.g., survival, health loss, costs) [12], and the macro economic consequences [14]. Methodological uncertainty is best dealt with by creating a reference case, i.e., the explicit list of methodological choices for model creation, so to allow for comparability between the choices authorities are presented with [15]. On the other hand, structural uncertainty refers to “uncertainty about the extent to which structural features of the model adequately capture the relevant characteristics of the disease and intervention being investigated” [12]. It includes decisions regarding what disease stages and transition possibilities to include in the model, how transition probabilities are derived, how missing data were dealt with, and if this transition is time- independent (like in our Markov model), or dynamic (changes over time) [16]. Structural uncertainty can be parameterized using different approaches (e.g., model averaging) [17]. Finally, parameter uncertainty refers to “uncertainty about the value for each parameter within the model, with respect to its true value” [3, 9, 18]. In this paper, we are only concerned with parameter uncertainty, and we focus on the maximum net benefit that can be gained when it is resolved completely, thus what is the EVPI.

### Chronic heart failure and telehealth

In 2012, 17.5 million people died from Cardio Vascular Diseases (CVDs), representing 31% of all global deaths [19]. Chronic Heart Failure (CHF) is one of the most prevalent CVDs, caused by age related changes in the cardiovascular system [20]. Telehealth solutions are proposed to tackle the challenge for healthcare systems of an increasing number of CHF patients [21, 22]. There were numerous studies on effectiveness of telehealth interventions in CHF management [23–28], a few studies on cost-effectiveness [29, 30], but not much was researched on the topic of VOI in CHF management via telehealth interventions. Given the burden of this disease and the uncertainty of the results of these studies, the value of forgone benefit is expected to be very large.

We were not able to find comparable studies on VOI in CHF management via telehealth in the Netherlands, but we did find on COPD in the Netherlands [10] and obesity in Switzerland [31]. Ramos et al. [32] performed a CEA with VOI for angiotensin inhibitors in CHF patients in the Netherlands, while McKenna performed two systematic reviews and economic analyses, on aldosterone antagonists [33] and external counterpulsation [34] in heart failure management. In our CEA [35] we compared our results with the results from Thokala et al. [30], the telehealth trial and cost-effectiveness analysis executed in the UK context. For their effective population of 54,779 heart failure patients the EVPI per patient at £20K/QALY was £826, while population EVPI at this threshold was £45,247,202 [36].

Previously, we conducted a cost-effectiveness analysis, using a cohort-level Markov model, comparing Home Telemonitoring (HTM) and Nurse Telephone Support (NTS) with Usual Care (UC) in CHF management in the Netherlands [35]. The cost-effectiveness analysis aimed to distinguish the healthcare intervention that would bring the highest net monetary/health benefit to the CHF patients in the Netherlands. Amongst other data sources, our model was mainly based on the clinical trial results from the Trans-European Network—Home-Care Management System (TEN–HMS) study [37]. The results from our CEA showed that both interventions are cost-effective in comparison to UC, considering the cost-effectiveness thresholds used by the decision makers in the Netherlands. The objective of this paper is to determine the value of resolving parametric uncertainty inherent in the current evidence and identify the impact of key parameters of the model—transition probabilities, utility generated, number of hospitalizations and ER visits, utilization of resources, and the disease severity—on the overall model parameter uncertainty. Thus, our study is an application of the VOI methodology to the problem of choosing between HTM and NTS over UC in CHF management in the Netherlands.

## Methods

### The Markov model

#### Structure of the model

**Fig 1:**
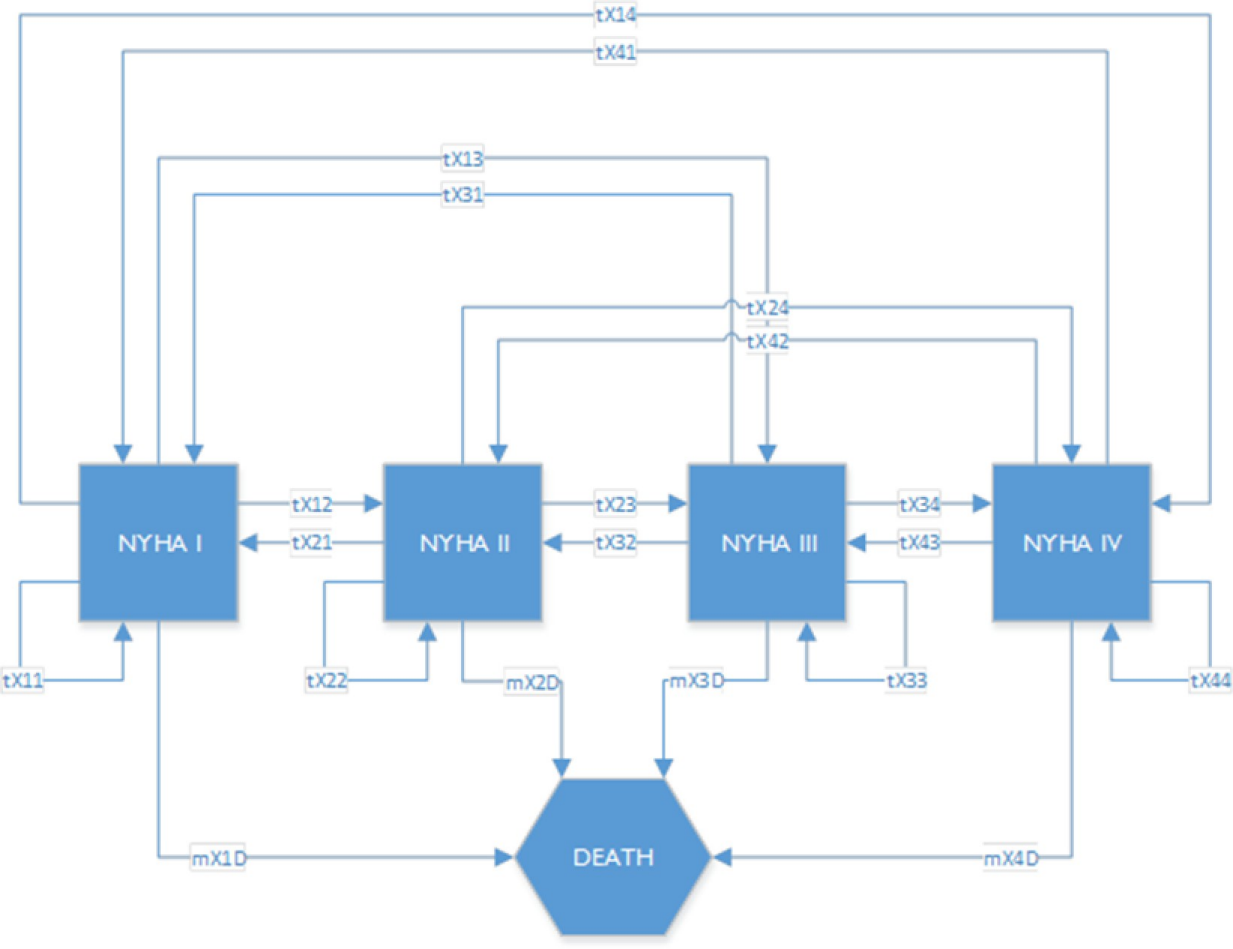
Diagrammatical representation of the model structure. *where X is "H" in HTM, "N" in NTS and "U" in UC.

The VOI analysis was applied to a cohort-level Markov model comparing the 20-year costs and effects in three interventions (i.e., HTM, NTS, and UC). A payer perspective was taken and the recommendations from the Dutch national pharmacoeconomic guideline [38] were followed. Details of the model have been published previously [35]. In brief, CHF patients were classified into four disease states of increasing severity based on the ability to walk and take care of themselves according to the New York Heart Association (NYHA) guidelines [39]. In prespecified time intervals of 4 months (i.e., Markov cycles), patients could stay in the same state, transition between disease states (from one NYHA class to any other class, e.g., from NYHA III to NYHA I, II, or IV class) or die, as shown in Fig 1. Time-variant, treatment-specific transition probabilities between the NYHA classes were derived from the observed transitions in the TEN-HMS study.

In the model, a cohort was tracked in time-independent manner (i.e., health state affiliations were averaged over the duration of the trial). From the total costs and the total QALYs generated in each health state, in each treatment arm, average costs and average QALYs per patient were calculated. Primary outcome of the model was the cost per QALYs gained per patient over 20 years. This time window corresponds to a life-time horizon since the mean age of a patient in TEN-HMS was 68 years in UC and 67 in HTM and NTS groups [37]. CHF is a severely progressive disease–average survival in Framingham Heart Study subjects was 1.7 years in men and 3.2 years in women [40]–thus all patients were expected to end-up in the death state at the end of the model’s time-horizon.

#### Model input parameters

Probabilistic input parameters of the model included transition probabilities between disease states, utilities associated with the disease states, number of hospitalizations (with and without ER visits), costs associated with the disease states (resource utilization including specialist/ general practitioner/ nurse contacts), and initial distribution of patients per NYHA group. Uncertainty around these parameters was considered simultaneously and each parameter was sampled from the corresponding distribution, independently. This was an important assumption in order to count the total costs and QALYs generated within all three intervention arms. We sampled the transition probabilities between disease states from Dirichlet distribution [41], and used beta distribution for sampling utilities, and uniform distribution for sampling the installment/equipment and service fee for HTM. Second-order Monte Carlo simulations (i.e., considering parameter uncertainty) were undertaken in which values were randomly drawn from these distributions.

We performed the VOI-analysis for two population sizes in the Netherlands—patients in all NYHA classes of severity, and patients in NYHA IV class only. All model parameters that are subject to parametric uncertainty were sampled from their characteristic distributions in the probabilistic sensitivity analysis and value of information analysis (e.g. all parameter except for discount rates and intervention acquisition costs). The all-NYHA-model starts with 19% of the patients in NYHA I class, 44% in NYHA II, 29% in NYHA III, and 8% in NYHA IV, as seen in the TEN-HMS study. Transition probabilities between NYHA disease states were based on the total number of transitions observed in the TEN-HMS clinical trial data, until the follow-up period of the trial. After the trial follow-up, same transition probabilities were assumed. Besides structural uncertainty of the model, there is an inherent uncertainty related to data imputation and how transition probabilities are estimated due to the missing data in the TEN-HMS database (due to stopping or missing entries). The mortality risk in each disease state was estimated by calculating the transition probability to death state from the observed deaths in the TEN-HMS trial.

Resource use was estimated from TEN-HMS trial data and unit costs (price level 2010) were obtained from the Dutch cost guideline [42] and updated to 2017 prices. We were interested in the number of contacts with nurses, general practitioners, specialists, and hospitalists, and the number of telephone calls with telenurses, emergency room visits, and hospital days. Resource use was tracked in time-independent manner (averaged over the duration of the trial) in each treatment arm. Utility values per disease state were based on the EQ5D-3L data from the TEN-HMS study, calculated by the Dutch utility weights. An overview of the model parameters can be seen in Table 1. The details of each input and the choices regarding them are discussed elaborately in the original paper [35].

**Table 1:** Overview of the model parameters.

Model Input	Source	Treatment specific?	Time invariant?	Distribution used in the PSA*
Initial distribution and transition probabilities among NYHA classes and death probabilities	TEN-HMS trial [37]	Yes	No	Dirichlet
Resource use (hospital, emergency room, nurse visit, GP, etc.) associated with each NYHA class	TEN-HMS trial [37]	Yes	Yes	Gamma
Utility values associated with each NYHA class	TEN-HMS trial [37] and Dutch utility weights [43]	No	Yes	Gamma
The unit cost for each type of resource use	Dutch cost guideline [44]	No	Yes	Gamma
Intervention/ comparator costs	Assumptions, treatment guidelines [45], TEN-HMS [37]	Yes	No	N/A

*PSA = Probabilistic Sensitivity Analysis

Fixed model parameters included discount rates for costs and effects, 4% and 1.5% respectively, as recommended by the Dutch National Health Care Institute [38]. Several publications are available in the literature advocating for differential discounting [46–48]. These are the discounting rates that were applied in the cost-effectiveness analysis. In the value of information analysis, discount rate was not applied in the per-patient EVPI and EVPPI calculations but in population level EVPI and EVPPI calculations, and the discount rate for costs was applied while calculating the effective population, which is multiplied by the per-patient EVPI/EVPPI values. Since net monetary benefit was used in the calculations, discount rate for costs was chosen over discount rate for effects.

For the VOI analyses, in contrast to our CEA [35], we modelled the increase in prevalence and incidence of CHF patients in the Netherlands according to the projections from the Dutch National Institute for Public Health and the Environment [49]. They estimated that approximately 1% of the adult Dutch population suffered from heart failure in 2012, i.e.,130,000 individuals, and that because of population aging the number of heart failure patients will increase to 195,000 individuals by 2025. The increment per year was thus estimated at 5,000 individuals, which we extrapolated till the end of our time-horizon in the model, starting with 160,000 individuals in 2018 to 255,000 in 2037. The effective population over 20 years was 2,841,567 (total number 4,150,000 discounted by 4%) in All NYHA classes of disease severity. For the number of NYHA IV patients we consulted the initial distribution of patients in the TEN-HMS trial (7.32%) and applied factor to the calculation. The effective population over 20 years In NYHA IV class analysis was 208,003 individuals.

### The value of information analysis

We run two analyses, one for all NYHA patients, and one for NYHA IV subgroup only, continuing the cost-effectiveness analyses from our previous work. Three factors determine the VOI [9]: 1) how cost-effective the technology appears given current or prior information, 2) the uncertainty surrounding cost-effectiveness (i.e., the distribution of the prior mean incremental net-benefit), and 3) consequences of decision error based on current information. The basic setup of a VOI problem is:

1. A decision variable or a set of decision variables, where one value can be selected for each decision variable;
2. State variables, some of which are uncertain;
3. A model relating independent state variable and decision variable values to outcomes (dependent state variables);
4. A payoff function over outcomes, and
5. A set of possible experiments which will reveal information about the state variables.

In the light of the discussion above, the mathematic formulation of the VOI analysis will be provided separately in the next chapter.

#### Expected value of perfect information

The expected value of perfect information is simply “the difference between the pay-off with perfect and current information” [10]. Payoff function in our decision problem context is the expected net monetary benefit, when a particular technology is chosen, in comparison to usual care.

Let *j* denote the decision variable, which is the decision of which technology to adopt. The selection of the UC is denoted when *j* = 0, the selection of the NTS is represented when *j* = 1, and the HTM is denoted when *j* = 2. Net monetary benefit (*NMB*_*j*_) is a commonly used payoff function in healthcare related VOI analyses, which is a random variable dependent on the selected technology *j*, given random input parameter realization *θ* in the sample space *Ω* (such as the transition probabilities, utility, resource use, or cost inputs) and for a given WTP threshold *λ*, it can be calculated as below with the help of the Markov model:

$$NMB_{j}(\theta ,\lambda )=\lambda \Delta E_{j}(\theta )-\Delta C_{j}(\theta )=\lambda (E_{j}(\theta )-E_{0}(\theta ))-(C_{j}(\theta )-C_{0}(\theta ))\;for\;j=0,1,2\;\mathrm{and}\;\mathrm{given}\;\theta \in \Omega$$

Given the existing evidence, there is uncertainty around the unknown parameters *θ*, and the optimal decision under the uncertainty would be to choose the intervention *j* that generates the maximum expected net benefit for a given accepted willingness to pay level *λ*.

$$\max_{j}(E_{\theta }(NMB_{j}(\theta |\lambda )))\;for\;\mathrm{given}\;\theta \in \Omega$$

When the uncertainty is resolved (under perfect information), the decision maker would know which value *θ* will take and for each *θ* the intervention that maximizes the net benefit for a given willingness to pay can be chosen: max_*j*_(*NMB*_*j*_(*θ*|*λ*)). However, since the true values are unknown, the expected value of a decision taken with perfect information should be found from the joint distribution of *θ*.

$$E_{\theta }(\max_{j}(NMB_{j}(\theta |\lambda )))$$

The overall EVPI per patient is simply the difference between the expected value of the decision made with perfect information about the uncertain parameters *θ* and the decision made based on the existing evidence.

$$EVPI_{per\;patient}=E_{\theta }(\max_{j}(NMB_{j}(\theta |\lambda )))-\max_{j}(E_{\theta }(NMB_{j}(\theta |\lambda )))$$

**Fig 2:**
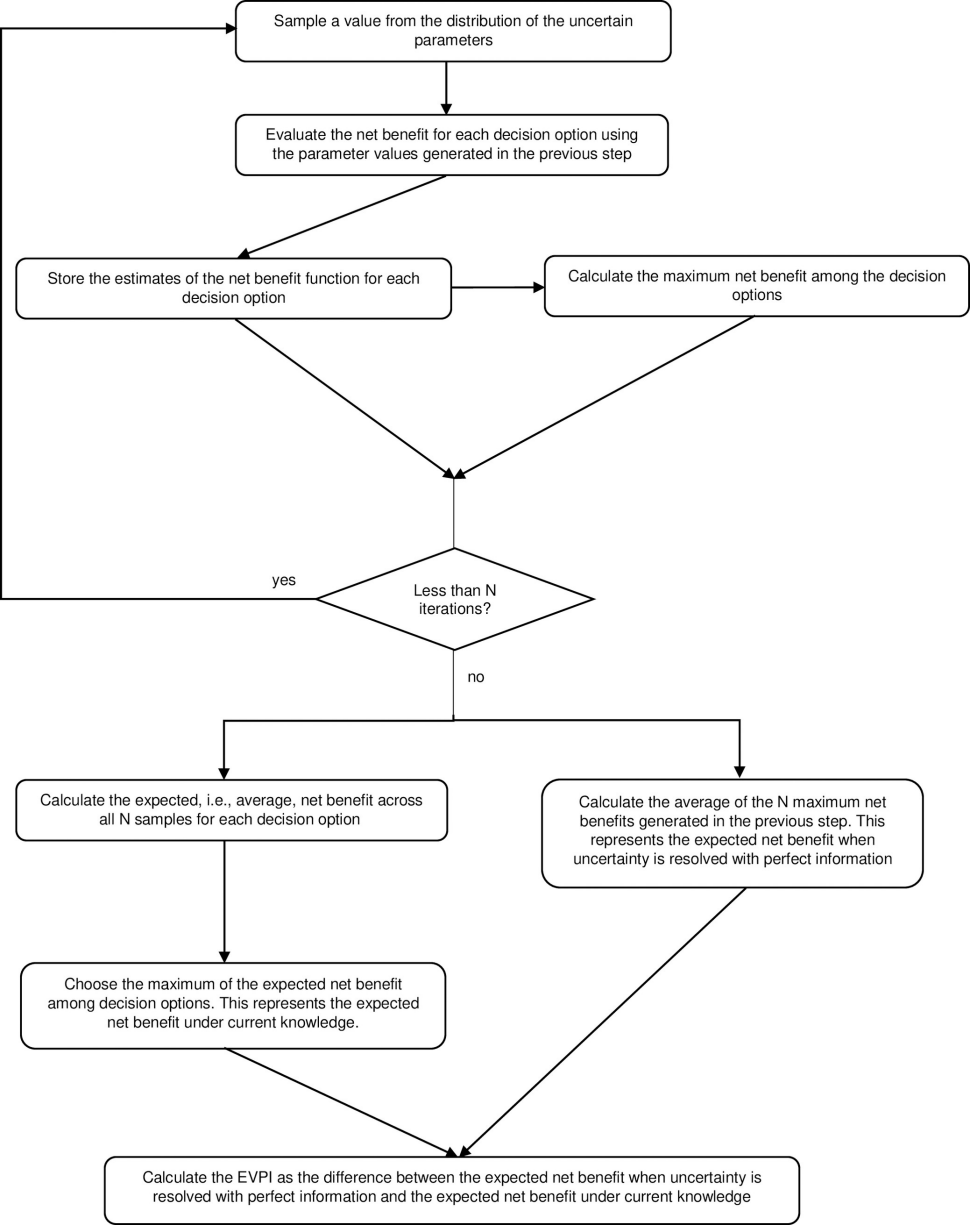
Single loop Monte Carlo scheme for computing overall individual EVPI. *adapted from Briggs et al. [9] and Oostenbrink et al. [10].

In our case, the expected value of a parameter is obtained following nonparametric approach, with the help of the Markov model. The uncertain parameters (*θ*) were sampled over 2,500 simulations. The EVPI analysis was performed using a one-level sampling algorithm, for an individual patient (Individual EVPI) as explained in Fig 2. After the individual EVPI is calculated, the population level EVPI (Population EVPI) can be also derived. It is the expected opportunity loss for the whole population that is to benefit from a technology of interest if a wrong decision is made due to parameter uncertainty. In order to obtain this, we need to understand the lifetime of the technology (in our model it is 20 years, although we are aware that there will be new generations of devices and services with improved effectiveness), the period over which information about the decision will be useful (till the end-of-life in our case), and the estimates of incidence over this period. The Population EVPI was calculated as:

$$PopulationEVPI=EVPI_{per\;patient}\sum_{t=1}^{T}\frac{I_{t}}{{\left(1+d\right)}^{t}}$$

*I*_*t*_: incidence in period *t*, *d*: discount rate, and *T* is the lifetime of the technology (number of time periods where the research would be useful).

Fig 2 depicts the process of calculating individual EVPI:

#### Expected value of partially perfect information

After exploring if potential further research on cost-effectiveness of HTM and NTS vs UC is cost-effective, i.e., EVPI is positive, we were interested in an indication of what type of additional evidence would be most valuable. The value of reducing the uncertainty surrounding a specific type/group of parameters of the model was found using a similar approach to the EVPI analysis. The expected value of perfect information for a parameter is the difference between the expected value with perfect and current information about that parameter group [9]. Due to computational reasons we approached the EVPPI analysis by conducting it first on a small number of groups of parameters. These groups were created based on the similarity of the parameters and a possibility to obtain these parameters from a single study. We grouped the cost-effectiveness model parameters into: 1) transition probabilities, 2) utilities, 3) hospitalizations and ER visits, 4) utilization of resources via nurse, general practitioner, medical specialist, and hospitalist contact or telephone call, and 5) initial distribution of patients per NYHA group. The grouping was performed according to parameter nature, reflecting a possible future study design method to inform the model [10].

**Fig 3:**
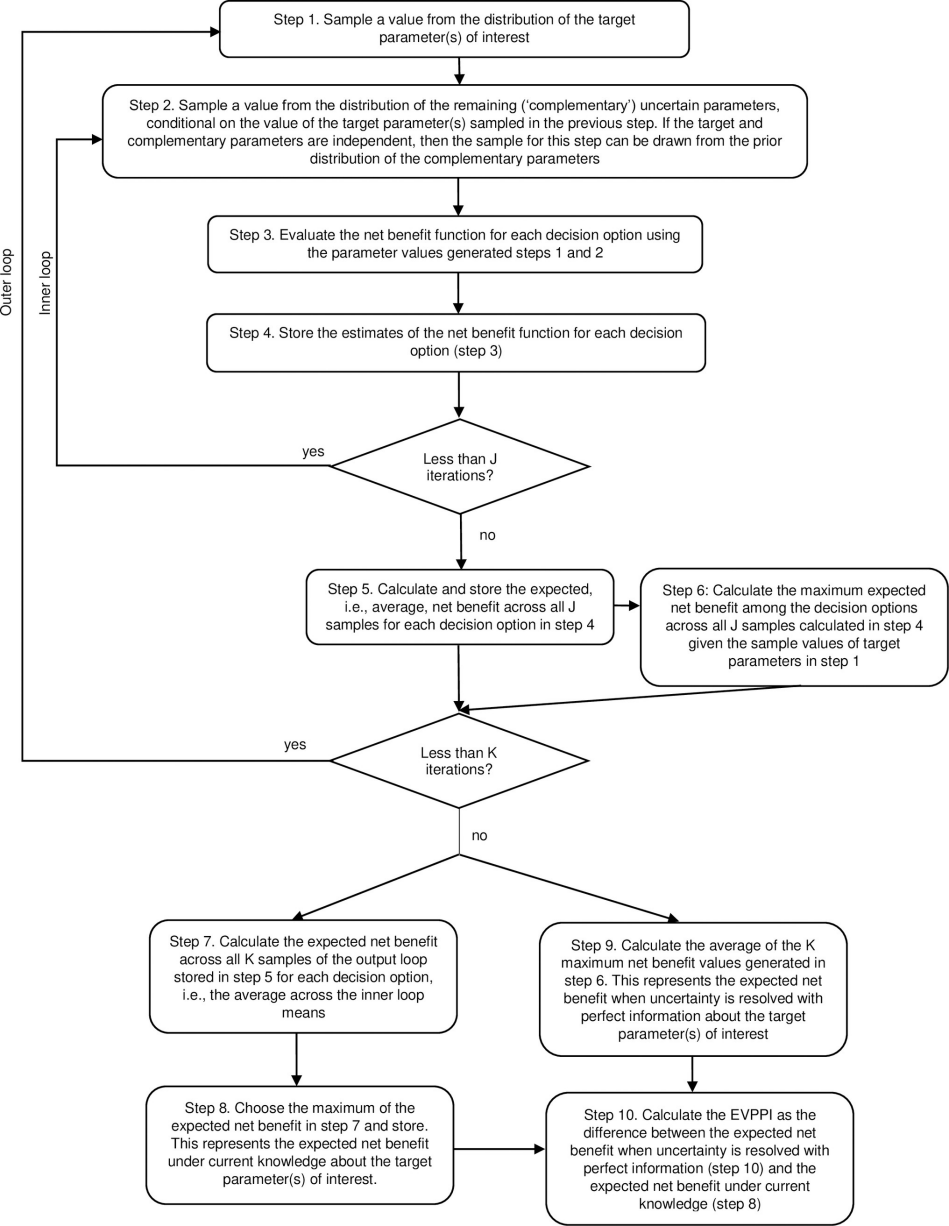
Sampling algorithm for the calculation of EVPPI with double loop (inner loop of size J and outer loop of size K). *adapted from Briggs et al. [9] and Oostenbrink et al. [10].

EVPPI was executed using a two-level sampling algorithm in which multiple simulations were performed for different values of a parameter of interest [6]. The two-level sampling algorithm used two nested levels of Monte Carlo sampling over the plausible ranges for both the parameter(s) of interest, and the remaining uncertain parameters. The two-level sampling algorithm that we have applied is outlined in Fig 3:

The double-loop algorithm requires substantial computation, however it was necessary since it does not depend on linearity assumptions as proposed in other single-loop approaches, e.g., SAVI [50]. This proves to be handy for models where the relationship between the parameters and the expected cost and outcomes is not linear, as it is in Markov models [9]. The inner and outer loop sizes (J and K) were determined iteratively, starting from simulation size of 500 for both inner and outer loops, and increasing by 500 until the EVPPI results did not change significantly (less than 1%). In all the group parameter EVPPI calculations both inner and outer loop sizes were smaller than or equal to 2,500. All analyses were performed in Excel in Microsoft Office 2016.

## Results

### Cost-effectiveness

**Fig 4:**
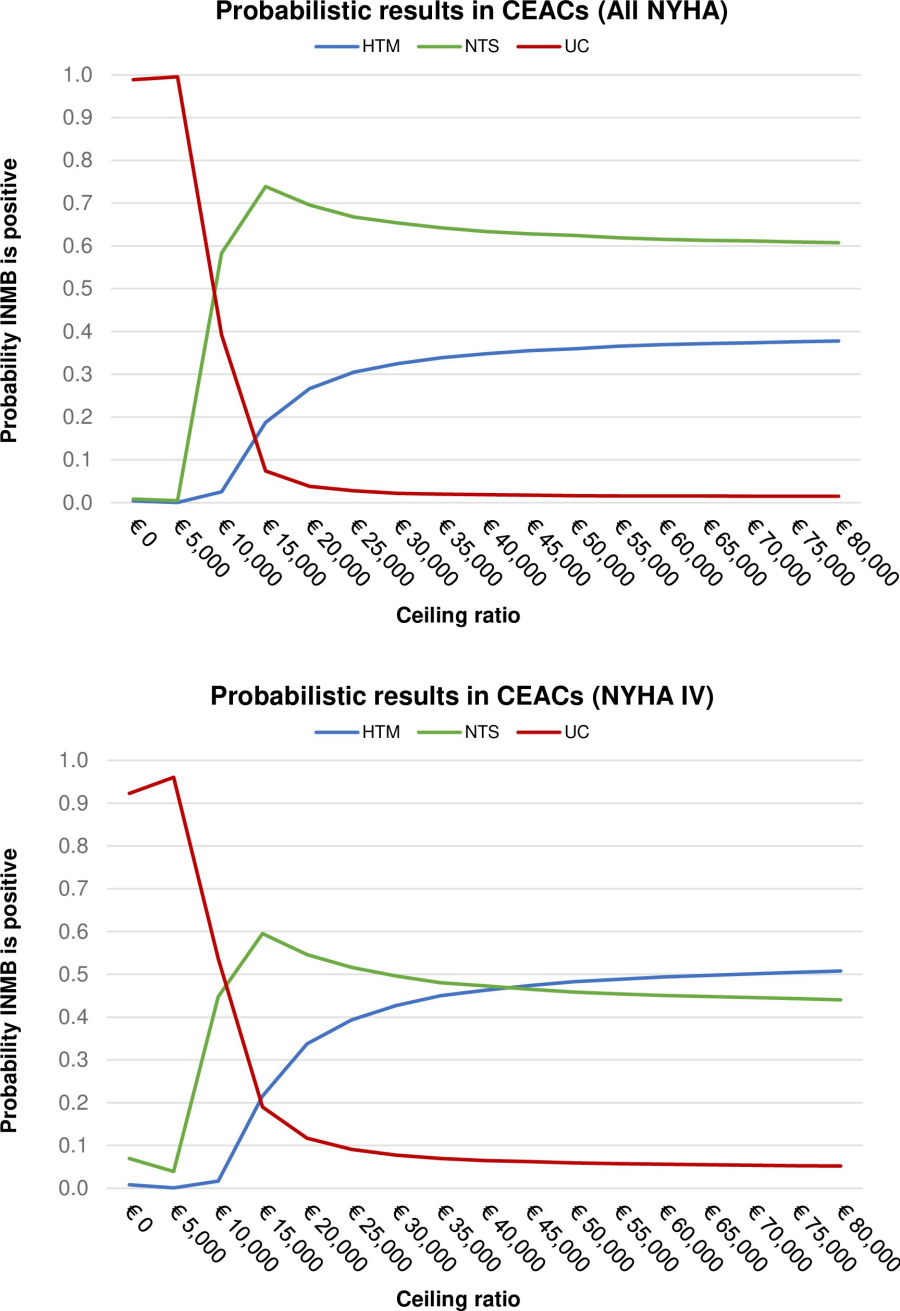
**Cost Effectiveness Acceptability Curves in a) All NYHA and b) NYHA IV analyses.** INMB = Incremental Net Monetary Benefit.

For all patients (All NYHA) the probability of HTM being cost-effective in comparison to NTS and UC increases as the WTP for additional health (i.e., QALY) or the threshold for cost-effectiveness increases (since the effectiveness difference is in favor of HTM), as shown in Fig 4A. The probability that HTM is the most cost-effective becomes higher than the probability that UC is the most cost-effective from WTP of approx. €14K and higher. There is no scenario where HTM is cost-effective in comparison to NTS, in all NYHA classes of patients combined (HTM is not a ‘part’ of the cost-effectiveness frontier).

In the subgroup analysis (NYHA IV), HTM is cost-effective in comparison to UC (and NTS) at WTP higher than approx. €40K/QALY. The CE horizon shows that UC should be preferred at approximately €9K/QALY and less, NTS from €9K to €40K, and HTM at higher WTP (Fig 4B).

### Individual EVPI

**Fig 5:**
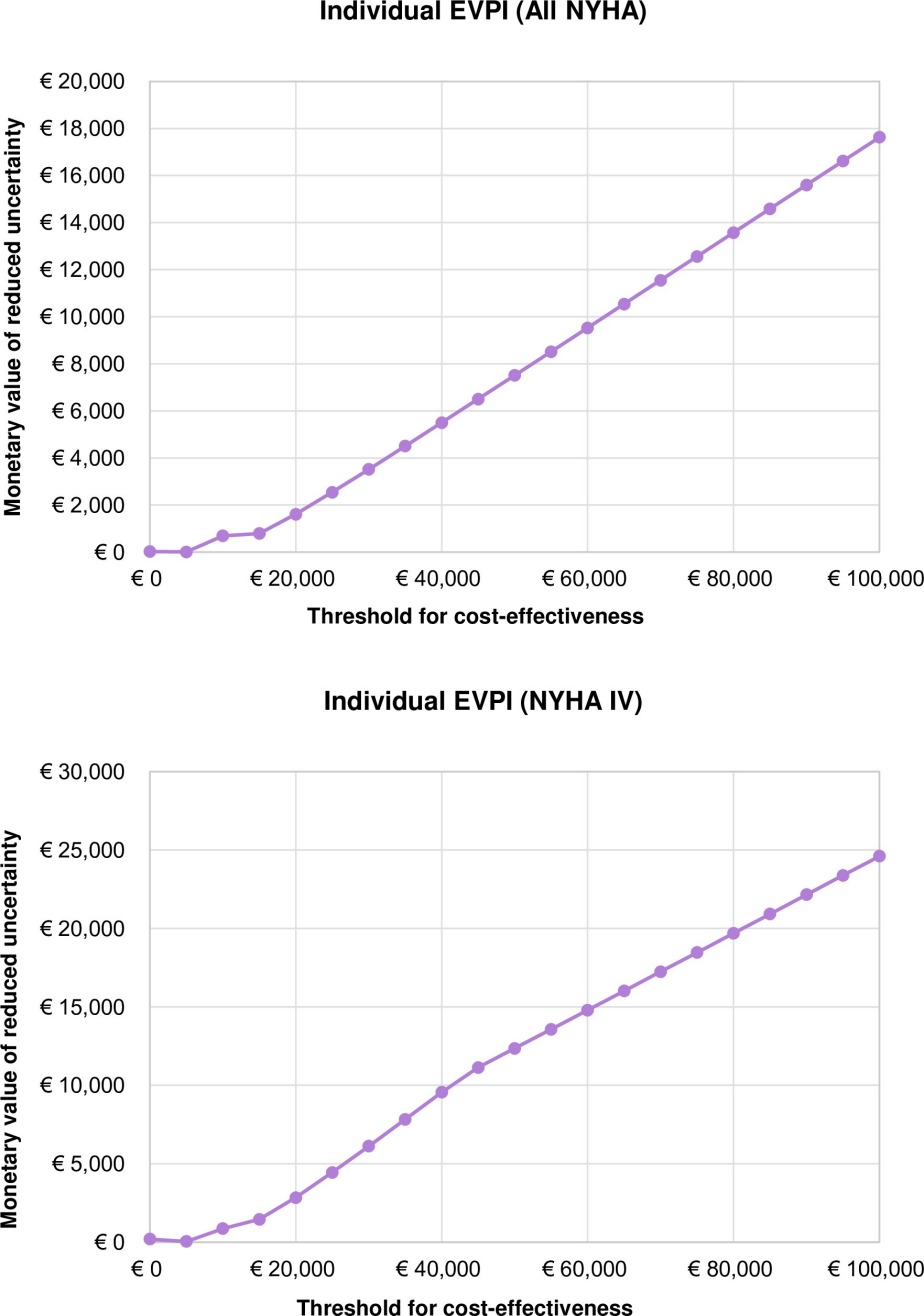
Individual Expected Value of Perfect Information in a) All NYHA and b) NYHA IV analyses.

The shape of the EVPI curve in Fig 5A is a representation of the average of the maximum net benefits with a perfect information, minus the maximum of the average expected net benefits across HTM, NTS, and UC. In All NYHA analysis there is a peak, i.e., a change in the slope of the EVPI curve, around the threshold values equal to the ICERs of each of the alternatives–NTS vs UC ICER €7,262 and HTM vs UC ICER €9,816. The decision uncertainty seams to linearly increase with the increase of the WTP because the probability of being cost-effective of the technologies compared with UC (HTM and NTS) seems to ‘plateau’ in Fig 4. The CEACs do not ‘meet each other’ at the high WTP thresholds, since probability of one of the technology options becoming the most cost-effective does not converge to 1 at higher WTP. Thus, the probability and the consequences of error raise, tending to increase EVPI, with increased WTP.

In NYHA IV analysis, the technology options on the CEAC frontier change in two points (Fig 4B), which is reflected by two ‘peaks’ in Fig 5B–at €10K and €45K/QALY. Here as well, the EVPI increase with WTP just as in All NYHA class analysis.

### Population EVPI

**Fig 6:**
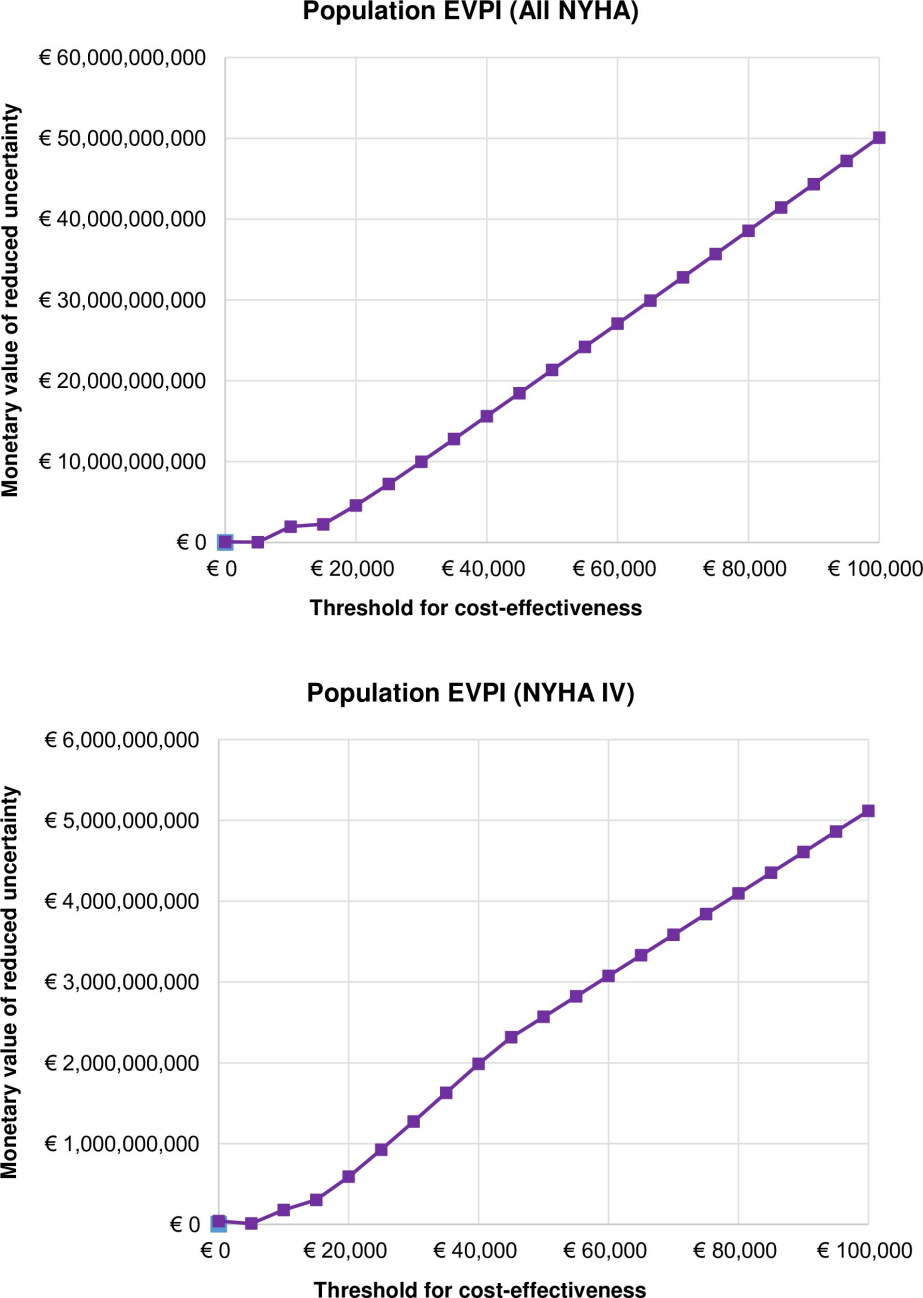
Population Expected Value of Perfect Information in a) All NYHA and b) NYHA IV analyses.

For our effective population of 2,841,567 patients in All NYHA stages of disease severity in the Netherlands, Fig 6A illustrates the Population EVPI over the next 20 years. If the cost for managing this population exceeds the expected costs of additional research, then it is potentially cost-effective to conduct further research on decision uncertainty. For example, the Netherlands pays €20K/QALY in management of CHF, and the Population EVPI at this WTP is more than €4.5B, implying that further research is highly cost-effective as opportunity costs are enormous. At lower values of the threshold, e.g., in the jurisdictions that pay only €5K/QALY, for the same population size the opportunity costs are slightly above €10M, and the new technology (i.e., HTM and NTS) should be rejected based on current evidence, and further research is required to support this decision, because the returns from further research cannot offset the costs.

In NYHA IV analysis, for the effective population of 208,003 patients over next 20 years, the Population EVPI at WTP of €20K/QALY is approx. €590M. Given the severity and prognosis of this disease stage, the authorities could potentially be willing to pay more per QALY generated, which will increase both the Population EVPI, but also the uncertainty of the decision to adopt HTM and NTS in the management of these patients.

### Individual EVPPI

**Fig 7:**
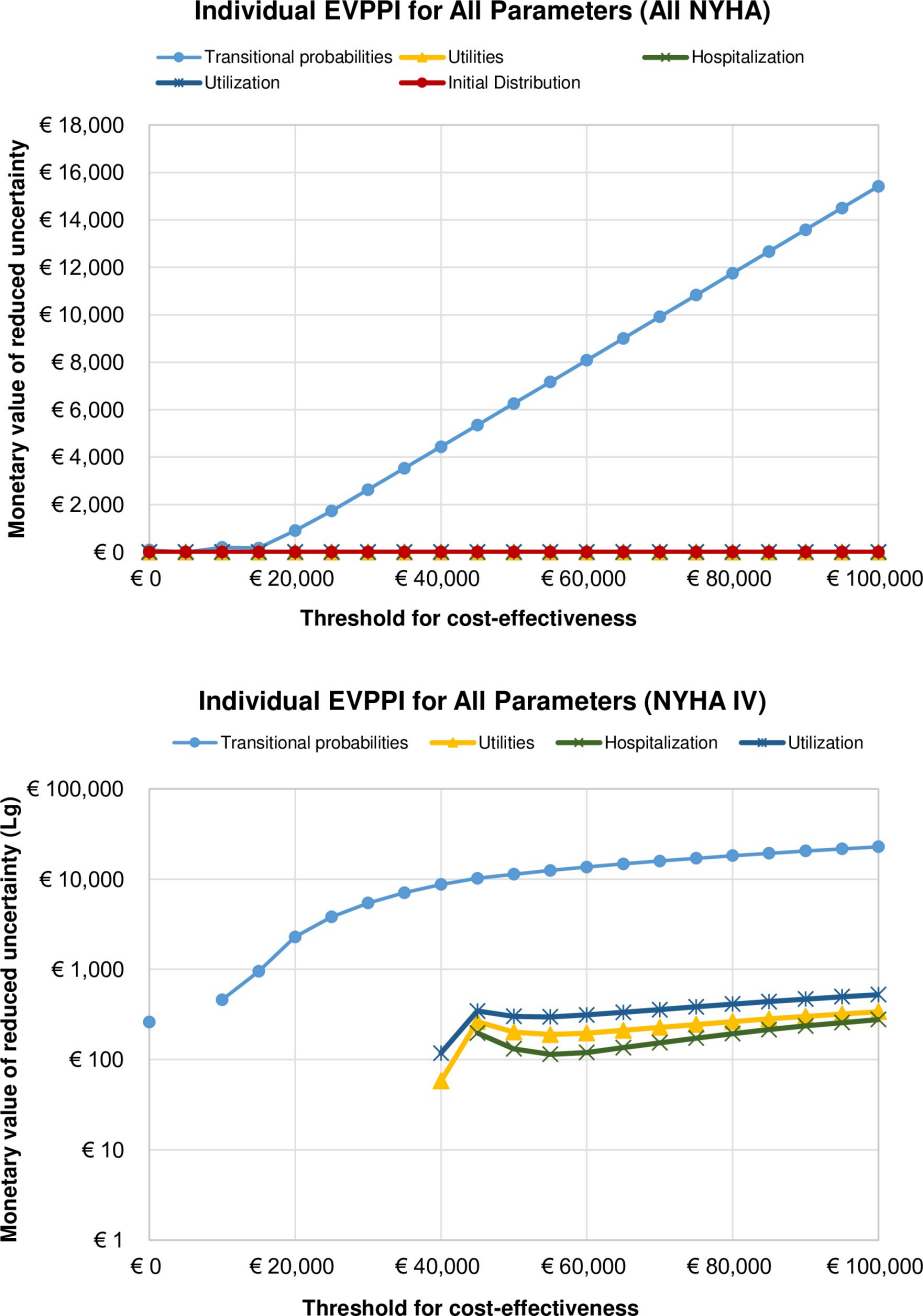
Individual Expected Value of Partially Perfect Information for all parameters in a) All NYHA and b) NYHA IV analyses.

Fig 7A shows the EVPPI for all groups of parameters in the model for one CHF patient (separate simulations were run, no correlation between sampled inputs)–transition probabilities, utilities, hospitalizations, and resource utilization (plus initial distribution of patients in All NYHA analysis). The results show that all decision uncertainty is attributable to uncertainties in transition probabilities, in All NYHA classes combined. The decision uncertainty increases linearly with the increase in WTP, which means that additional research on transition probabilities for HTM and NTS in CHF management is cost-effective and needed. However, the EVPPI for utilities, hospitalization, utilization, and initial distribution is zero at all WTPs, thus resolving parametric uncertainty from these parameters does not seem to add any value or reduce decision uncertainty in All NYHA classes combined.

In the NYHA IV analysis, there are again two ‘peaks’ (at €10K and €45K/QALY) for transition probabilities, corresponding to the technology change on the CEAC frontier, with even higher net monetary benefit value than in All NYHA for given WTP values (approx. €10K in NYHA IV vs approx. €5K in All NYHA) indicating that uncertainty around transitions from and to NYHA IV group drive the overall uncertainty around transition probabilities in our model. The peaks at €10K cannot be seen in Fig 7B due to the log scale of the y-axis. For utilities, hospitalizations, and resource use we observe nonzero EVPPI values from WTP higher than €40K, having a hunch at €45K and increasing in a linear fashion after €60K.

### Population EVPPI

**Fig 8:**
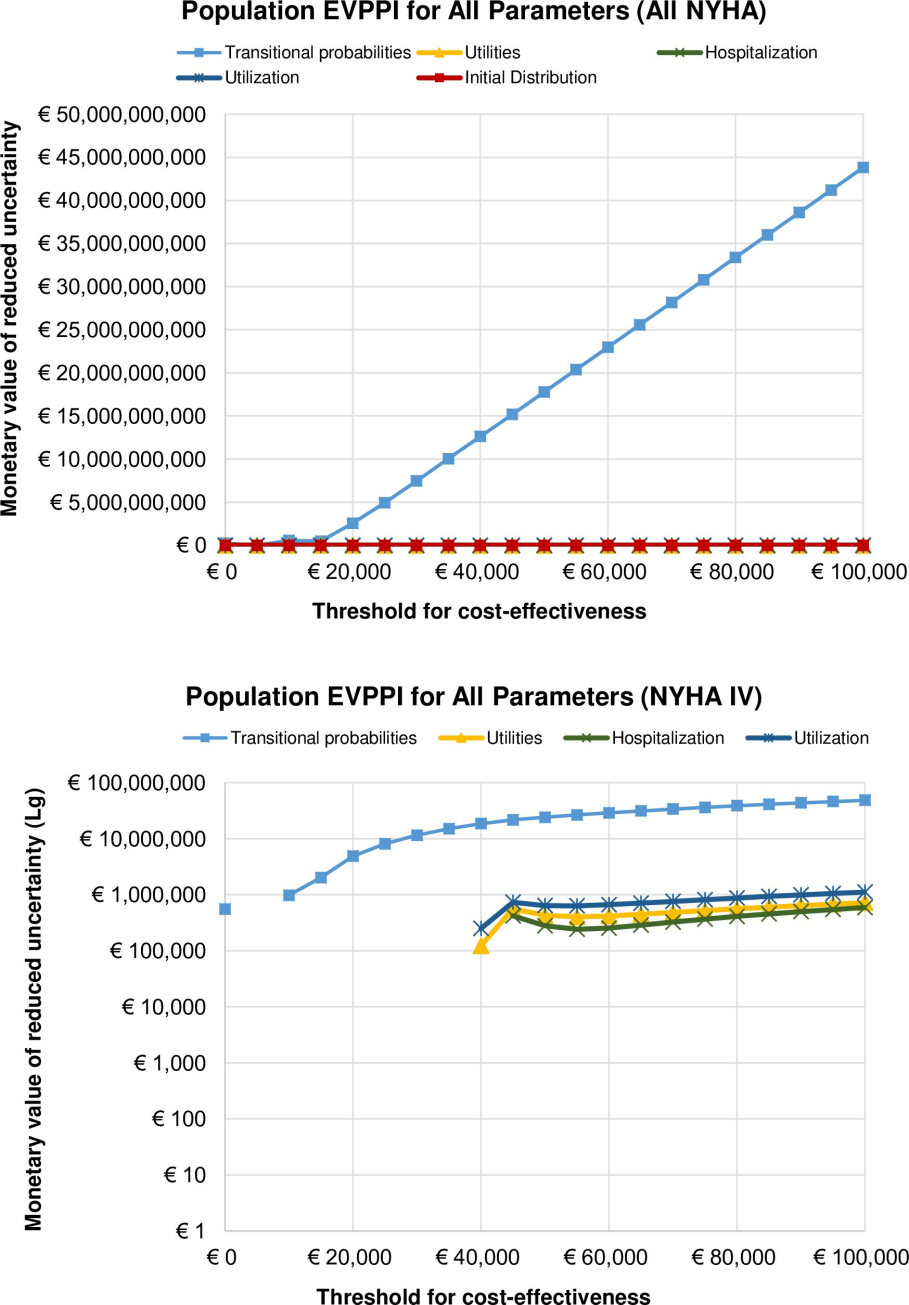
Population Expected Value of Partially Perfect Information for all parameters in a) All NYHA and b) NYHA IV analyses.

Fig 8A presents the Population EVPPI results where simulations for all five groups of parameters are to be found in one graph (again, separate simulations were run) for a population of 2,841,567 patients. It is evident that future research should focus on transition probabilities, i.e., disease progression in both HTM and NTS interventions in management of CHF. It seems that at the WTP threshold of €20K/QALY the expected value of partially perfect information for a future (20 years) CHF population in the Netherlands is approx. €2.5B. For a population in All NYHA classes there is no gain in understanding the uncertainty around other parameters except transition probabilities in our model. The opportunity loss for the future (20 years) population of NYHA IV CHF patients in the Netherlands at €20K/QALY is approx. €4.8M, and slowly rises with increase of WTP (Fig 8B).

## Discussion

In this modelling study, we were interested in knowing the decision uncertainty regarding telehealth implementation in CHF management in the Netherlands. The decision variable was which telehealth system should the Netherlands adopt–none, NTS, or HTM. The potentially uncertain parameters included transition probabilities, utility values, staff utilization, and hospitalizations and ER visits. The payoff was defined as net monetary benefit at the individual level, and at the national level as the sum of the payoff over individuals.

The decision to implement new technologies will always be uncertain, and that uncertainty is conditional on the cost-effectiveness of new technologies. If the decision based on current information is ‘wrong’ there will be consequences in terms of opportunity loss, i.e., monetary and health benefits forgone. The opportunity loss can be calculated from the estimates of probability and cost of error. This is the expected cost of uncertainty. In other words, “the expected cost of uncertainty can be interpreted as the expected value of perfect information, as perfect information can eliminate the possibility of making the wrong decision” [9]. Analytical techniques such as value of information and portfolio decision can be used to prioritize research investments as they seek to quantify the expected return of alternative research efforts relative to their cost [51]. Keisler et al. [52] performed a literature review, from years 1990–2011, and found a substantial increase in published papers utilizing VOI methodology, particularly in the medical field.

Results of the Individual EVPI analysis (Fig 5) show that where there is more uncertainty (i.e., greater variance in incremental net-benefit), the probability of error will increase and expected opportunity loss and EVPI will be higher. This is because the variance of net monetary benefits increases with the increase of WTP threshold, and as we compare three options (i.e., HTM, NTS, and UC) the variability and uncertainty are greater than when comparing two alternatives. When the threshold for cost-effectiveness is low, the technology is not expected to be cost-effective and additional information is unlikely to change that decision (EVPI is low). In case of All NYHA and NYHA IV the EVPI increases with the threshold because the decision uncertainty (probability of error) increases and the consequences of decision error (opportunity loss) are valued more highly.

For the Population EVPI (Fig 6), i.e., the expected perfect information for the total number of patients that can benefit from HTM and NTS, the value of information is expressed over the model time horizon. As telehealth technology will not last 20 years, we added replacement costs every 5 years. However, we expect the effectiveness of both HTM and NTS to remain over the next two decades, or even increase. Thus, our effectiveness estimation can be considered conservative. The EVPI associated with future patients is discounted at a rate of 4% and runs in billions of euros for CHF patients in the Netherlands. The population EVPI can be the first step in identifying whether future research is cost-effective, and which research venues are worthwhile [9].

With our modelling exercise we have found that the future research on uncertainty surrounding implementation of HTM and NTS is cost-effective. We were then interested in knowing what additional evidence would be most valuable in reducing this uncertainty. After all, the VOI analysis performed here was based on a fairly old data originating from the clinical trial (TEN-HMS) that took place between January 2000 and July 2002. The value of reducing uncertainty of particular parameters in our model was established using a similar approach to the EVPI analysis. The Individual EVPPI was found by “taking the maximum expected net-benefit given perfect information only about the parameter(s) of interest (calculating expected net benefits over all the other uncertain parameters in the model) and then calculating the mean of all the possible values of the parameters of interest” [9]. The EVPPI analysis showed that the only group of parameters that have a substantial impact on the decision uncertainty are transition probabilities, in both All NYHA and NYHA IV analyses, and that the future research should concern disease progression. The optimal research designs to apply would be randomized control trials, or prospective and retrospective studies. In NYHA IV analysis some uncertainty was found for utilities, number of hospitalizations, and resource use at WTP thresholds higher than €45K.

Limitations of our study were plentiful. In the model there is a substantial amount of structural uncertainty, especially in terms of how transition probabilities are derived, assumptions on time dependence, and data imputation. From the previous appraisals, we know that sometimes structural uncertainty cannot be parameterized and might contribute most to the decision uncertainty [53–55]. Also, utility and resource use costs are state-dependent and not time/treatment-dependent. The assumptions from the original modelling study remained [35] and were supplemented with the new ones: a) presuming that HTM and NTS will have same costs and effectiveness over the next 20 years, b) the increase of CHF population in the Netherlands by 5,000 each years for the next 20 years, c) applying the 4% discount rate, d) grouping the parameters assuming independence. Individual parameters when considered in isolation might not resolve at maximum values, to have an impact on the NMBs, but when grouped together they might resolve in such a way to have a significant impact on differences in net benefits and change the decision. EVPPI for individual parameter can be zero, but if grouped there is a possibility to have a substantial impact [9], and thus our grouping according to possible future methods for evidence gathering is biased. There is also a possibility of correlation between the parameters, and grouping will preserve the correlation structure, if done properly. If the correlation exists, there is a possibility that the EVPPI for the group of parameters will be greater than the EVPI of the same group, or even the EVPI for the decision itself [9], which was our case with transition probabilities. To remedy this situation, we run 2,500 simulations for this parameter group, and 1,000 for all others. We left the Expected Value of Sample Information (EVSI), the continuation of the VOI analysis for calculation of optimum sample size and allocation rates in randomized clinical trials [6], for future research.

The conclusion of our VOI analysis is that further research into the transition state probabilities seem to decrease the decision uncertainty the most, among the analyzed parameter groups. Almost all of the input parameters (e.g. utilities and resource utilization) are not treatment-specific but are dependent on NYHA-class. In the model it was assumed that only the transition probabilities among NYHA classes and death are contingent on the treatment choice. The difference in cost, QALY, and life-year outcomes from different treatment choices are instrumentalized by the differences between transition probabilities (among NYHA classes and death). Therefore, the uncertainty of this group of parameters contribute to the decision uncertainty the most. Since in each NYHA class a patient would be at risk of death, transitions among NYHA classes cannot be observed separately, thus, in a VOI problem these should be analyzed together. This, and the finding that value of information about the transition probabilities turns out to be high might be relevant to other applications in healthcare. The case presented here might be useful as a methodological exercise for other real-world problems.

The decision to adopt HTM and NTS in management of CHF in the Netherlands ultimately relies on cost-effectiveness of those technologies, uncertainty (variability of NMBs) surrounding cost-effectiveness, and cost of the decision error. The authorities must reach a decision if further research is warranted, or the current evidence is ‘good enough’ for reimbursement of these technologies. Claxton et al. [56] argue that in addition to approval or rejection, the authorities should also consider ‘only in research’ or ‘approval with research’. The benefits of immediate access might exceed the value of future research, and the decision should not be solely based on expected net benefit [57]. Immediate approval can provide an incentive to a manufacturer, consequently lowering cost of technology, and thus improving cost-effectiveness of HTM and NTS. Rejecting a promising technology in healthcare based on cost-effectiveness prevents us from learning about its performance. That is why decision making in reimbursement of medical devices is so difficult and should also consider learning curve effects, incremental device innovation, investment and irrecoverable costs, and dynamic pricing [58].

Our research shows that the decision uncertainty in adopting HTM and NTS in CHF management in the Netherlands lies predominantly with the transition probabilities (i.e., the change of a NYHA class in a Markov cycle), and more effort should be given to understanding the dynamics of the disease progression. Results of our modelling exercise show that the cost of uncertainty for all NYHA patients in the Netherlands in the next 20 years amounts to €4.5B at WTP of €20K/QALY. This renders future research in telehealth for the management of CHF in the Netherlands cost-effective, and the return-on-investment substantial.
